# A New Belief Entropy to Measure Uncertainty of Basic Probability Assignments Based on Belief Function and Plausibility Function

**DOI:** 10.3390/e20110842

**Published:** 2018-11-03

**Authors:** Lipeng Pan, Yong Deng

**Affiliations:** Institute of Fundamental and Frontier Science, University of Electronic Science and Technology of China, Chengdu 610054, China

**Keywords:** Dempster–Shafer (D–S) theory, belief entropy, Deng Entropy, measurement uncertainty, probability interval, belief function

## Abstract

How to measure the uncertainty of the basic probability assignment (BPA) function is an open issue in Dempster–Shafer (D–S) theory. The main work of this paper is to propose a new belief entropy, which is mainly used to measure the uncertainty of BPA. The proposed belief entropy is based on Deng entropy and probability interval consisting of lower and upper probabilities. In addition, under certain conditions, it can be transformed into Shannon entropy. Numerical examples are used to illustrate the efficiency of the new belief entropy in measurement uncertainty.

## 1. Introduction

With the sharply growing interest in data fusion, the evidence theory, also known as the Dempster–Shafer (D–S) theory [1], which was first presented by Dempster [2] and then developed by Shafer [3], has aroused great concern for its effectiveness in modeling and fusing uncertain information [4]. D–S theory assigns probabilities to the power set of events [5], so it has advantages of dealing with uncertainty and unknown problems. In addition, it has wide applications, such as sensors’ network analysis [6], classification and clustering [7,8,9], decision-making [10,11,12], knowledge reasoning [13,14], risk assessment and evaluation [10,15], and others [9,11,16,17,18].

The D–S theory is used to combine belief functions [2,19,20]. However, in D–S theory, there is an open issue on how to measure the uncertainty of belief functions [2,5,21,22]. Uncertainty plays a significant role in some fields since it is the foundation and prerequisite to quantitatively study the questions [3,23,24,25]. Shannon entropy has basically resolved the uncertainty of probability theory [26], which is widely used in many application systems [27,28,29]. Inspired by ideas, many scientists are devoted to studying uncertainty of belief function [30]. So far, there are some methods of uncertainties in belief function [31]. We classify these methods according to additivity [32,33]. Deng entropy [34] and Tsallis [35] entropy do not satisfy the additivity, which are non-extended entropy. In addition, Yager’s specificity measure [31], Hartley entropy [36], Korner’s specificity definition [37], Höhle confusion measure [38], discord measure [39] and conflict measure [40] satisfy additivity. Generally speaking, the measures can reduce to Shannon’s entropy under certain conditions. However, in recent studies, there is an important discovery that belief function theory is not a successful generalization of probability theory [3,41]. The basic probability assignment (BPA) function is transformed into probability distribution through conversion, which results in the loss of information. Hence, it is unreasonable that uncertainty of belief functions was calculated by the evolution of Shannon entropy. Therefore, it is very desirable to define a new way of measuring uncertainty to avoid the loss of information. Based on that, many people have made some attempts in the field, Deng [34] has presented Deng entropy to simplify the calculation of uncertainty of BPAs by considering total non-specificity and discord simultaneously without the conversion from BPA to probability. Recently, the probability interval in BPA has aroused wide attention because it is also a key factor for uncertainty. Yang and Han [41] have defined a distance-based total uncertainty measure for BPA based on probability interval. Deng et al. [42] have improved this measure to avoid counter-intuitive results caused by it. They overcome some shortcomings of traditional measurement; however, the uncertainty of those methods is inconsistent with Shannon entropy when BPA is degenerated to probability distribution.

In this paper, we analyze the uncertainty of BPA based on intervals which contain more information than probability. We propose new belief entropy by combining probability interval and Deng Entropy’s idea, which can degenerate Shannon entropy when there is probability distribution. Thus, our proposed method can effectively measure uncertainty in BPA and probability distribution. Since there is no switch between BPA and probability distribution, it can overcome these limitations in traditional measures. Thus, it is feasible to define an uncertainty measure for a BPA based on probability interval.

The paper is organized as follows. Basics of D–S evidence theory for BPA are briefly introduced in Section 2. Section 3 presents and existing uncertainty measures and new belief entropy of BPA. Some important examples are described in Section 4 in order to illustrate the efficiency of the new belief entropy. Finally, this paper is concluded in Section 5.

## 2. Preliminaries

In this section, some preliminaries are briefly introduced.

### D–S Evidence Theory

Some basic definitions of D–S theory are briefly introduced [2,3]:

A set of hypotheses Θ is the exhaustive hypotheses of variable θ [43]. The elements are mutually exclusive in Θ [44]. Then, Θ is called the frame of discernment, defined as follows [2,3]:

(1) $$\Theta =\{\theta_{1},\theta_{2},\cdots ,\theta_{i},\cdots ,\theta_{N}\}.$$

The power set of Θ is denoted by $2^{\Theta }$ [45], and

(2) $$2^{\Theta }=\left\{\emptyset ,\left\{\theta_{1}\right\},\cdots ,\left\{\theta_{N}\right\},\left\{\theta_{1},\theta_{2}\right\},\cdots ,\left\{\theta_{1},\theta_{2},\cdots ,\theta_{i}\right\},\cdots ,\Theta \right\},$$

where ∅ is an empty set [46].

A BPA function *m* is a mapping of $2^{\Theta }$ to a probability interval [0,1], formally defined by [2,3]:

(3) $$m:\;2^{\Theta }\to \left[0,1\right],$$

which satisfies the following conditions [47]:

(4) $$\begin{array}{c} m\left(\emptyset \right)=0\;\sum_{A\in 2^{\Theta }}m(A)=1\;0\leq m\left(A\right)\leq 1\;A\in 2^{\Theta }. \end{array}$$

The mass m(A) represents how strongly the evidence supports *A*.

The belief function (Bel) is a mapping from set $2^{\theta }$ to [0, 1] and satisfied:

(5) $$Bel\left(A\right)=\sum_{B\subseteq A}m\left(B\right).$$

When BPA is $m\left(A\right)=\left\{\begin{array}{cc} 1 & A=\Theta \\ 0 & A\neq \Theta \end{array}\right\}$, the Bel is the simplest, $Bel\left(m\right)=\left\{\begin{array}{cc} 1 & A=\Theta \\ 0 & A\neq \Theta \end{array}\right\}$, this bel is called a vacuous belief function which is suitable for situations without any evidence.

The plausibility function (Pl): $2^{\theta }\to \left[0,1\right]$, and satisfied:

(6) $$Pl\left(A\right)=\sum_{B\cap A\neq \phi }m\left(B\right)=1-Bel\left(\bar{A}\right).$$

The Pl indicates the degree to which is not suspected.

As can be seen from the above, ∀A⊆Θ,$Bel\left(A\right)$ < $Pl\left(A\right)$, Bel(A),Pl(A) are respectively the lower and upper limits of A, namely [Bel(A), Pl(A)], which indicates uncertain interval for A.

For the same evidence, the different BPAs come from the different evidence resources. The Dempster’s combination rule can be used to obtain the combined evidence [2,48]:

(7) $$\left\{\begin{array}{c} m(\emptyset )=0\\ m\left(A\right)=\frac{\sum_{B⋂C=A}m_{1}\left(B\right)m_{2}\left(C\right)}{1-K}, \end{array}\right.$$

where $K=\sum_{B⋂C=\emptyset }m_{1}\left(B\right)m_{2}\left(C\right)$. It is remarkable that, if K>1, the Dempster’s rules can not apply to two BPAs.

## 3. Uncertainty Measures for Belief Structures

### 3.1. Existing Uncertainty Measures for Belief Structures

There are many methods to handle uncertainty [49]. In 1948, Shannon pointed out: “Information is used to eliminate random uncertainty” and proposed the concept of “information entropy” (using the concept of entropy in thermodynamics) to solve the problem of information measurement [50]. The concept of entropy is derived from physics [50,51]; it has been a measure of uncertainty and disorder [52]. A system with higher uncertainty has greater entropy, which also contains more information [11].

The Shannon entropy *H* is derived as [26,53]:

(8) $$H=-\sum_{i=1}^{N}p_{i}log_{b}p_{i},$$

where *N* is the number of basic states in a system, and $p_{i}$ is the probability of state *i* appears satisfying $\sum_{i=1}^{N}p_{i}=1$.

Shannon entropy plays a key role in handling a basic probability problem, and there are some limitations of Shannon entropy [42]. The concept of entropy in the framework of D–S theory is an open issue. Many researchers have extended many measured functions based on it, such as:

Dubois and Prade. Dubois and Prade weighted Hartley entropy of BPA was shown [54]:

(9) $$H_{dp}\left(m\right)=-\sum_{A\subseteq 2^{\Theta }}m\left(A\right)log\left(|A|\right).$$

Höhle. One of the earlier confusion measures for D–S theory was due to Höhle [38]:

(10) $$H_{o}\left(A\right)=-\sum_{A\subseteq 2^{\Theta }}m\left(A\right)log\left(Bel\left(A\right)\right).$$

Yager. Dissonance measure of BPA was defined by Yager, as follows [31]:

(11) $$H_{y}\left(m\right)=-\sum_{A\subseteq 2^{\Theta }}m\left(A\right)logPl\left(A\right).$$

Klir and Ramer. Another discord measure of BPA was defined by Klir and Ramer, as follows [39]:

(12) $$H_{kr}=-\sum_{A\subseteq \Theta }m\left(A\right)log\sum_{B\subseteq \Theta }m\left(B\right)\frac{\left|A\cap B\right|}{B}.$$

Klir and Parviz. Klir and Parviz defined entropy [40]:

(13) $$H_{kp}\left(m\right)=-\sum_{A\subseteq \Theta }m\left(A\right)log\sum_{B\subseteq \Theta }m\left(B\right)\frac{\left|A\cap B\right|}{\left|A\right|}.$$

George and Pal. George and Pal suggested a definition of conflict measure [55]:

(14) $$H_{gp}\left(m\right)=\sum_{A\subseteq \Theta }m\left(A\right)\sum_{B\subseteq \Theta }m\left(B\right)\left|1-\frac{A\cap B}{A\cup B}\right|.$$

It can clearly be seen that these methods are all based on the Shannon entropy. There are also some documents that give a detailed introduction to these functions [49,56,57], and these entropies have their own basic properties, such as consistency with D–S theory semantics, non-negativity, probability consistency, etc. and later Deng proposed the concept of Deng Entropy [34], which is a new function of measuring uncertainty. The Deng entropy is described as follows [34]:

(15) $$H_{d}\left(m\right)=-\sum_{A\subseteq 2^{\Theta }}m\left(A\right)log\frac{m(A)}{2^{\left|A\right|}-1},$$

where |A| is the cardinality of A. As the above, Deng Entropy is very similar to Shannon Entropy, but Deng Entropy uses $2^{\left|A\right|}-1$ to deal with the BPA of multifocal elements, which is more advantageous than Shannon Entropy. In addition, additivity and boundary are expanded.

### 3.2. The New Belief Entropy

In D–S theory, the probability interval [Bel(A),Pl(A)] can be obtained more information based on the basic probability assigned to each focal element. In this article, we use the probability interval to extend new methods of measuring uncertainty, as follows:

(16) $$H_{bel}\left(m\right)=-\sum_{A\subseteq 2^{\Theta }}\frac{Bel(A)+Pl(A)}{2}log\frac{Bel(A)+Pl(A)}{2(2^{\left|A\right|}-1)}.$$

As mentioned, this probability interval whose lower and upper bounds are the Bel and the Pl, respectively [58,59]. For a probability distribution, there are some advantages, such as discord and non-specificity [60]. Moreover, central values of probability interval can be used to compare uncertainty. At length, we all know that cardinality of every BPA is very important for the measurement of uncertainty. Hence, the new belief entropy which considers Deng entropy and the interval probability can better measure the uncertainty of BPA. In addition, according to the the literature of Kirl and Lewis [32], Kirl [33], the basic properties of the new belief entropy are explored as follows:

$\left(P1\right)$ consistency with DS theory semantics: The new entropy is consistent with D–S theory semantics. Thus, it satisfies the consistency with D–S theory semantics property.

$\left(P2\right)$ non-negativity: We know that $0<\frac{\left\{Ble\left(x\right)+Pl\left(x\right)\right\}}{2}<1$, thus, $H_{bel}\left(m\right)>0$. For $H_{bel}\left(m\right)=0$ to hold, only if $m\left\{\left(x\right)\right\}=1$, $H_{bel}\left(m\right)=0$ if and only if m is Bayesian. Thus, new entropy satisfies the non-negativity property.

$\left(P3\right)$ probability consistency: If *m* is Bayesian, then $m\left(x\right)=Ble\left(x\right)=Pl\left(x\right)$, for all xϵX. Thus, new entropy satisfies the probability consistency property.

$\left(P4\right)$ subadditivity: To check that new entropy does not verify the subadditivity property, we consider the following example:

Let X×Y be the product space of the sets $X=\left\{x_{1},x_{2},x_{3}\right\}$ and $Y=\left\{y_{1},y_{2}\right\}$. We have that the marginal BPAs on X×Y with masses

$m\left(\left\{z_{11},z_{12},z_{21}\right\}\right)=0.5,m\left(\left\{z_{31},z_{32}\right\}\right)=0.1,m\left(\left\{z_{21}\right\}\right)=0.1,m\left(X\times Y\right)=0.3,$ where $z_{ij}=\left(x_{i},y_{j}\right)$. We have that the marginal BPAs on X×Y are the following ones: $m_{1}$ and $m_{2}$, respectively

$$m_{1}\left(x_{1},x_{2}\right)=0.5,m_{1}\left(x_{3}\right)=0.1,m_{1}\left(x_{2}\right)=0.1,m_{1}\left(X\right)=0.3,$$

$$m_{2}\left(y_{2}\right)=0.1,m_{2}\left(Y\right)=0.9.$$

Thus:

$$Bel\left(x_{1},x_{2}\right)=0.6,Pl\left(x_{1},x_{2}\right)=0.9,Bel\left(x_{3}\right)=0.1,Pl\left(x_{3}\right)=0.4,$$

$$Bel\left(x_{2}\right)=0.1,Pl\left(x_{2}\right)=0.9,Bel\left(X\right)=1,Pl\left(X\right)=1,$$

$$Bel\left(y_{1}\right)=0.1,Pl\left(y_{1}\right)=1,Bel\left(Y\right)=1,Pl\left(Y\right)=1,$$

$$Bel\left(\left\{z_{11},z_{12},z_{21}\right\}\right)=0.6,Pl\left(\left\{z_{11},z_{12},z_{21}\right\}\right)=0.9,Bel\left(\left\{z_{31},z_{32}\right\}\right)=0.1,Pl\left(\left\{z_{31},z_{32}\right\}\right)=0.4,$$

$$Bel\left(\left\{z_{21}\right\}\right)=0.1,Pl\left(\left\{z_{21}\right\}\right)=0.9,Bel\left(X\times Y\right)=1,Pl\left(X\times Y\right)=1,$$

$$H_{bel}\left(m_{1}\right)+H_{bel}\left(m_{2}\right)=7.36669,H_{bel}\left(m\right)=9.79031.$$

Obviously,$H_{bel}\left(m\right)>H_{bel}\left(m_{1}\right)+H_{bel}\left(m_{2}\right)$, and the subadditivity property is not satisfied.

$\left(P5\right)$ additivity properties: The new entropy is also non-additive. It is easy to check, in general, that $2^{mn}-1\neq \left(2^{m}-1\right)\times \left(2^{n}-1\right)$. We can use the following counter example to prove it in a more direct way:

Using the symbol of the previous example. Let X×Y be the product space of the sets $X=\left\{x_{1},x_{2},x_{3}\right\}$ and $Y=\left\{y_{1},y_{2}\right\}$. We have that the marginal BPAs on X×Y are the following ones: $m_{1}$ and $m_{2}$, respectively:

$$m_{1}\left(x_{1},x_{2}\right)=0.5,m_{1}\left(x_{3}\right)=0.1,m_{1}\left(x_{2}\right)=0.1,m_{1}\left(X\right)=0.3,$$

$$m_{2}\left(y_{2}\right)=0.1,m_{2}\left(Y\right)=0.9.$$

Now, we build the following BPA $m^{{}^{\prime }}=m_{1}\times m_{2}$ on X×Y (the marginal BPAs of $m^{{}^{\prime }}$ are $m_{1}$ and $m_{2}$; and they are noninteractive). The BPA $m^{{}^{\prime }}$ has the following masses:

$$m^{{}^{\prime }}\left(\left\{z_{11},z_{12},z_{21}\right\}\right)=0.5,m^{{}^{\prime }}\left(\left\{z_{31},z_{32}\right\}\right)=0.1,m^{{}^{\prime }}\left(\left\{z_{21}\right\}\right)=0.1,m^{{}^{\prime }}\left(X\times Y\right)=0.3,$$

where $z_{ij}=\left(x_{i},y_{j}\right)$. Thus,

$$H_{bel}\left(m_{1}\right)+H_{bel}\left(m_{2}\right)=7.36669,H_{bel}\left(m^{{}^{\prime }}\right)=9.79031.$$

Again, $H_{bel}\left(m^{{}^{\prime }}\right)>H_{bel}\left(m_{1}\right)+H_{bel}\left(m_{2}\right)$, and the additivity property is not satisfied by the new belief entropy. Therefore, the new entropy satisfies the consistency with D–S theory semantics, non-negativity, probability, and does not satisfy additivity properties, sub-additives. Therefore, the basic properties of some current entropies are given in Table 1.

In addition, BPA reflects more information than probability distribution in D–S theory. There is a classic example as follows:

Assume in a test that there are 32 students participating in a course examination. The teacher has scores of these students. A teacher is only allowed to answer “Yes” or “No” to any questions, in order to know who is (are) the top student who gets (get) the highest score(s). How many times do we need to ask at most? Assume that the time is t, and it is easy to answer the problem through calculating the information volume by using information entropy $t=log_{2}32=5$ However, when we have been told that there are two students tied for first. The entropy is still 5? In this case, how many times do we need to ask at most to know who are the first ONES? In this case, obviously t≥5.

It can be seen from this example that the uncertainty of BPA is greater than the probability distribution. Thus, the uncertain measure boundary of probability distribution should be extended.

On the other hand, it can be found from recent research that the application of Tsallis entropy as non-additive entropy is more and more extensive [61]. The additivity entropy is a special case of the non-additivity entropy. As a result, the two requirements above, namely boundary and additivity, should be improved.

## 4. Numerical Experimental

In this section, some numerical examples are used to illustrate the application of our approach.

### 4.1. Example 1

Assume that the frame of discernment is Θ={A} and we are given a BPA from a sensor as m({A})=1. Thus, we can calculate the Bel and Pl by Equations (5) and (6):

$$\begin{array}{c} Bel(A)=1,Pl(A)=1. \end{array}$$

Moreover, their classical Shannon entropy and the new belief entropy was calculated as follows:

$$\begin{array}{c} H\left(m\right)=H\left(m\right)=-1\times log1=0,H_{bel}\left(m\right)=-1\times log1=0. \end{array}$$

From above, we can conclude that the new belief entropy will retrograde the Shannon entropy if the frame of discernment has a single element. Under these circumstances, there is no uncertainty:

### 4.2. Example 2

Given that the frame of discernment is $\Theta =\left\{\theta_{1},\theta_{2},\theta_{3},\theta_{4}\right\}$, for a mass function $m\left(\theta_{1}\right)=m\left(\theta_{2}\right)=m\left(\theta_{3}\right)=m\left(\theta_{4}\right)=\frac{1}{4}$, then:

$$\begin{array}{c} Bel\left(\theta_{1}\right)=Bel\left(\theta_{2}\right)=Bel\left(\theta_{3}\right)=Bel\left(\theta_{4}\right)=\frac{1}{4}, \end{array}$$

$$\begin{array}{c} Pl\left(\theta_{1}\right)=Pl\left(\theta_{2}\right)=Pl\left(\theta_{3}\right)=Pl\left(\theta_{4}\right)=\frac{1}{4}, \end{array}$$

$$\begin{array}{c} H\left(m\right)=-\frac{1}{4}\times log\frac{1}{4}+-\frac{1}{4}\times log\frac{1}{4}+-\frac{1}{4}\times log\frac{1}{4}+-\frac{1}{4}\times log\frac{1}{4}=2, \end{array}$$

$$\begin{array}{c} H_{bel}\left(m\right)=-\frac{\frac{1}{4}+\frac{1}{4}}{2}\times log\frac{\frac{1}{4}+\frac{1}{4}}{2\times (2^{1}-1)}+-\frac{\frac{1}{4}+\frac{1}{4}}{2}\times log\frac{\frac{1}{4}+\frac{1}{4}}{2\times (2^{1}-1)}\\ +-\frac{\frac{1}{4}+\frac{1}{4}}{2}\times log\frac{\frac{1}{4}+\frac{1}{4}}{2\times (2^{1}-1)}+-\frac{\frac{1}{4}+\frac{1}{4}}{2}\times log\frac{\frac{1}{4}+\frac{1}{4}}{2\times (2^{1}-1)}=2. \end{array}$$

Obviously, the Shannon entropy and the new belief Entropy are the same when dealing with a mass function of a single element. It further demonstrates the feasibility of the new belief entropy.

### 4.3. Example 3

Given a frame of discernment $\Theta =\left\{\theta_{1},\theta_{2},\theta_{3},\theta_{4}\right\}$, for a mass function $m(\theta_{1},\theta_{2},\theta_{3},\theta_{4})=1$, then:

$$\begin{array}{c} H_{bel}\left(m\right)=-\frac{1+1}{2}\times log\frac{1+1}{2^{4}-1}=3.90689. \end{array}$$

In comparison of Example 2, the uncertainty of Example 3 is bigger than Example 2. Because m(Θ)=1 contains more information, that is to say, the mass function is totally unknown for system. However, for Example 2, the probability distribution contains less information than m(Θ)=1. Therefore, the result is reasonable.

### 4.4. Example 4

Given a framework $\Theta =\left\{\theta_{1},\theta_{2},\theta_{3},\theta_{4}\right\}$, for a mass function $m\left(\theta_{1}\right)=\frac{1}{4},m\left(\theta_{2}\right)=\frac{1}{3},m\left(\theta_{1},\theta_{2}\right)=\frac{1}{6},m\left(\theta_{3}\right)=\frac{1}{6},m\left(\theta_{4}\right)=\frac{1}{12}$, whose $H_{bel}\left(m\right)$ are calculated as follows:

$$Bel\left(\theta_{1}\right)=\frac{1}{4},Pl\left(\theta_{1}\right)=\frac{5}{12},$$

$$Bel\left(\theta_{2}\right)=\frac{1}{3},Pl\left(\theta_{2}\right)=\frac{1}{2},$$

$$Bel\left(\theta_{1},\theta_{2}\right)=\frac{3}{4},Pl\left(\theta_{1},\theta_{2}\right)=\frac{3}{4},$$

$$Bel\left(\theta_{3}\right)=\frac{1}{6},Pl\left(\theta_{3}\right)=\frac{1}{6},$$

$$Bel\left(\theta_{4}\right)=\frac{1}{12},Pl\left(\theta_{4}\right)=\frac{1}{12},$$

$$\begin{array}{c} H_{bel}=-\frac{\frac{1}{4}+\frac{5}{12}}{2}log\frac{\frac{1}{4}+\frac{5}{12}}{2(2^{1}-1)}+\left(-\frac{\frac{1}{3}+\frac{1}{2}}{2}log\frac{\frac{1}{3}+\frac{1}{2}}{2(2^{1}-1)}\right)+\left(-\frac{\frac{3}{4}+\frac{3}{4}}{2}log\frac{\frac{3}{4}+\frac{3}{4}}{2(2^{2}-1)}\right)+\\ \left(-\frac{\frac{1}{6}+\frac{1}{6}}{2}log\frac{\frac{1}{6}+\frac{1}{6}}{2(2^{1}-1)}\right)+\left(-\frac{\frac{1}{12}+\frac{1}{12}}{2}log\frac{\frac{1}{12}+\frac{1}{12}}{2(2^{1}-1)}\right)=3.28415. \end{array}$$

### 4.5. Example 5

Given a framework $\Theta =\left\{\theta_{1},\theta_{2},\theta_{3},\theta_{4}\right\}$, for a mass function $m(\theta_{1})=\frac{1}{4},m(\theta_{2})=\frac{1}{3},m(\theta_{3})=\frac{1}{6},m(\theta_{1},\theta_{2},\theta_{3})=\frac{1}{6},m(\theta_{4})=\frac{1}{12}$, whose $H_{bel}\left(m\right)$ are calculated as follows:

$$Bel\left(\theta_{1}\right)=\frac{1}{4},Pl\left(\theta_{1}\right)=\frac{5}{12},$$

$$Bel\left(\theta_{2}\right)=\frac{1}{3},Pl\left(\theta_{2}\right)=\frac{1}{2},$$

$$Bel\left(\theta_{3}\right)=\frac{1}{6},Pl\left(\theta_{3}\right)=\frac{1}{3},$$

$$Bel\left(\theta_{1},\theta_{2},\theta_{3}\right)=\frac{11}{12},Pl\left(\theta_{1},\theta_{2},\theta_{3}\right)=\frac{11}{12},$$

$$Bel\left(\theta_{4}\right)=\frac{1}{12},Pl\left(\theta_{4}\right)=\frac{1}{12},$$

$$\begin{array}{c} H_{bel}=-\frac{\frac{1}{4}+\frac{5}{12}}{2}log\frac{\frac{1}{4}+\frac{5}{12}}{2(2^{1}-1)}+\left(-\frac{\frac{1}{3}+\frac{1}{2}}{2}log\frac{\frac{1}{3}+\frac{1}{2}}{2(2^{1}-1)}\right)+\left(-\frac{\frac{1}{6}+\frac{1}{3}}{2}log\frac{\frac{1}{6}+\frac{1}{3}}{2(2^{1}-1)}\right)+\\ \left(-\frac{\frac{11}{12}+\frac{11}{12}}{2}log\frac{\frac{11}{12}+\frac{11}{12}}{2(2^{3}-1)}\right)+\left(-\frac{\frac{1}{12}+\frac{1}{12}}{2}log\frac{\frac{1}{12}+\frac{1}{12}}{2(2^{1}-1)}\right)=3.31977. \end{array}$$

These are the two examples we randomly choose. It can be seen that $m\left(\theta_{1},\theta_{2},\theta_{3}\right)=\frac{1}{6}$ in Example 5 is one more element than $m\left(\theta_{1},\theta_{2}\right)=\frac{1}{6}$ in Example 4, which will cause the entropy of Example 5 to be larger than the entropy of Example 4. This result is reasonable.

### 4.6. Example 6

Given a frame of discernment $\Theta =\left\{\theta_{1},\theta_{2},\cdots ,\theta_{N}\right\}$, there are three special cases of mass function as follows:

$$\begin{array}{c} m_{1}\left(A\right)=\frac{2^{A}-1}{\sum_{B\subseteq \Theta }2^{\left|B\right|}-1},A,B\subseteq \Theta , \end{array}$$

$$\begin{array}{c} m_{2}\left(\Theta \right)=1, \end{array}$$

$$\begin{array}{c} m_{3}\left(\theta_{1},\theta_{2},\cdots ,\theta_{N}\right)=\frac{1}{N}. \end{array}$$

**Figure 1:**
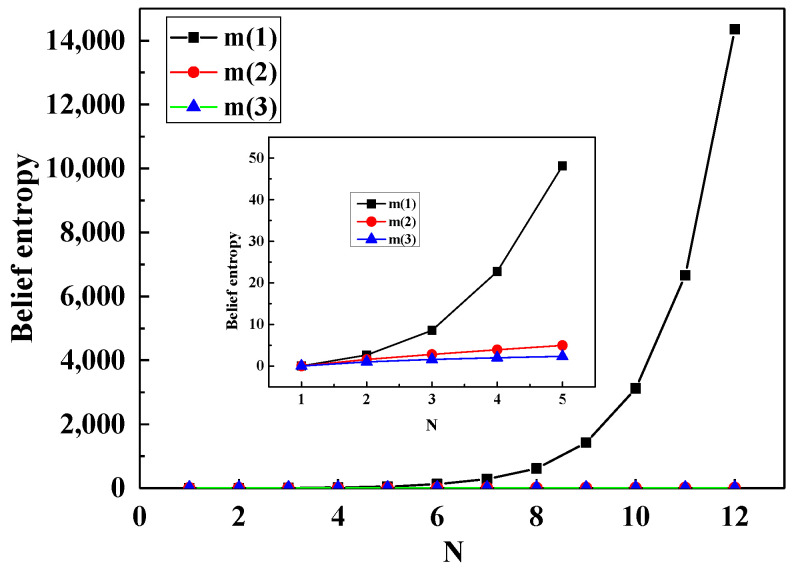
New belief entropy as a function of size of frame of discernment in three types of BPA.

Their associated new belief entropy accompanied by the change of N of $m_{1}$, $m_{2}$, $m_{3}$ was shown in Figure 1. It can be seen from Figure 1 that, with the increase of *N*, the mass function $m_{1}$ has the maximum uncertainty which grows very fast, while the Bayesian function $m_{3}$ has the minimal uncertainty. By comparison, we know that the $m_{1}$ represents more information than $m_{2}$, $m_{3}$.

### 4.7. Example 7

Given a frame with 15 elements identifying A, the elements are from 1 to 15, and the basic mass function is as follows:

$$\begin{array}{c} m(3,4,5)=0.05,m(7)=0.05,m(A)=0.8,m(\Theta )=0.1. \end{array}$$

**Figure 2:**
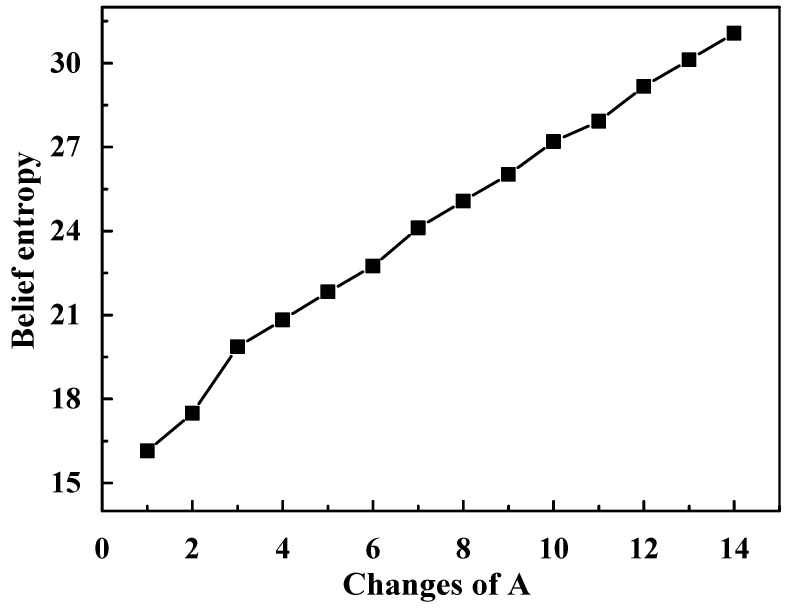
New belief entropy as a function of changes of A.

Table 2 reflects the trend of the new belief entropy when A changes, which can be seen from Figure 2. The calculation results show that, as the elements in A continue to increase, the uncertainty of BPA also increases. It is rational that there is more uncertainty with more elements.

**Figure 3:**
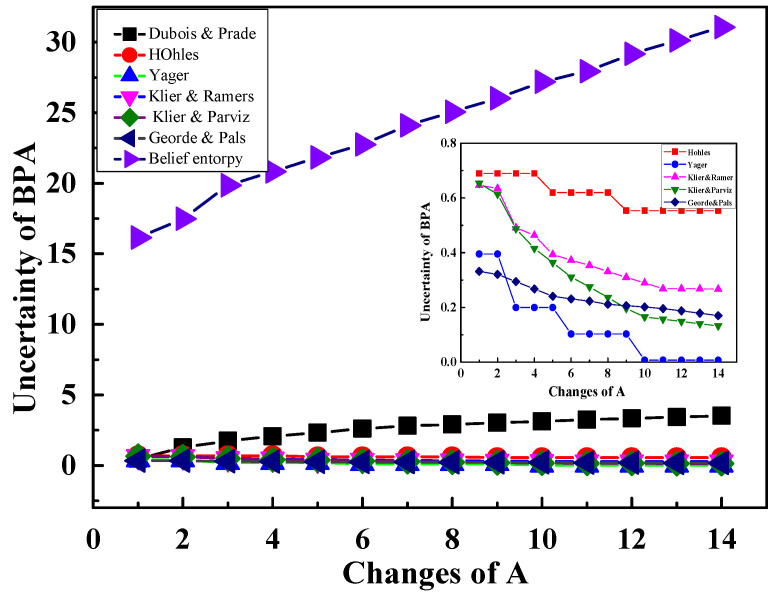
Different measurement of uncertainty with changes of A of BPA.

Furthermore, in the experiment, we also used different methods to measure the uncertainty of the BPA, such as Dubois and Prade’s weighted Hartley entropy [54], Höhle’s confusion measure [38], Yager’s dissonance measure [31], Klir and Ramer’s discord [39], Klir and Parviz’s strife [40], and George and Pal’s conflict measure [55]. The experimental results are shown in Figure 3. It is obvious that only the new belief entropy and Dubois and Prade’s weighted Hartley entropy increase constantly with the rise of the size of A. On the contrary, it can be seen from the insert in Figure 3 that the uncertainty obtained by other methods are reducing or changing irregularly when the A increases, which is obviously unreasonable. Therefore, the uncertainty of the new entropy in BPA Measurements are effective. Moreover, there are some differences between the new belief entropy and Dubois and Prade’s weighted Hartley entropy, and Dubois and Prade’s weighted Hartley entropy is not degenerate into Shannon entropy when the mass function is defined as a probability distribution. Therefore, the new belief entropy is a reasonable measure among these given uncertainty measures, which combine probability interval and cardinality of multiple elements of the BPA, and it is also more flexible.

## 5. Conclusions

Shannon entropy can effectively measure uncertainty of probability distribution. For the BPA, although many methods have appeared to measure the uncertainty, there is an open issue. The main work of this paper is to propose a new belief entropy without the conversion from BPA to probability based on probability interval and cardinality of multiple elements of BPA. The new belief entropy would have more uncertainty than other entropies, and the boundary and additivity have been improved. The new belief entropy is a generalization of the Shannon entropy, which can degenerate into the Shannon entropy when the BPA is a probability distribution. Moreover, some numerical examples are used to show the efficiency of the proposed new belief entropy.

## Figures and Tables

**Table 1:** The above table is extracted from the article of Jiroušek and Shenoy [49], For the sake of comparison, the last line adds the property of the new entropy.

Definition	Cons.with D–S	Non-neg	Prob.cons	Additivity	Subadd
Höhle	yes	no	yes	yes	no
Smets.	yes	no	no	yes	no
Yager	yes	no	yes	yes	no
Nguyen	yes	no	yes	yes	no
Dubois–Prade	yes	no	no	yes	yes
Lamata–Moral	yes	yes	yes	yes	no
Klir–Ramer	yes	yes	yes	yes	no
Klir–Parviz	yes	yes	yes	yes	no
Pal et al	yes	yes	yes	yes	no
Maeda–Ichihashi	no	no	yes	yes	yes
Harmanec–Klir	no	no	yes	yes	yes
Abellán–Moral	no	no	yes	yes	yes
Jousselme et al	no	yes	yes	yes	no
Pouly et al	no	yes	yes	yes	no
Deng	yes	yes	yes	no	no
New entropy	yes	yes	yes	no	no

**Table 2:** New belief entropy when A changes.

Cases	New Belief Entropy
A = {1}	16.1443
A = {1, 2}	17.4916
A = {1, 2, 3}	19.8608
A = {1, 2, 3, 4}	20.8229
A = {1, 2, ⋯, 5}	21.8314
A = {1, 2, ⋯, 6}	22.7521
A = {1, 2, ⋯, 7}	24.1131
A = {1, 2, ⋯, 8}	25.0685
A = {1, 2, ⋯, 9}	26.0212
A = {1, 2, ⋯, 10}	27.1947
A = {1, 2, ⋯, 11}	27.9232
A = {1, 2, ⋯, 12}	29.1370
A = {1, 2, ⋯, 13}	30.1231
A = {1, 2, ⋯, 14}	31.0732
